# Complete chloroplast genomes of three wild perennial *Hordeum* species from Central Asia: genome structure, mutation hotspot, phylogenetic relationships, and comparative analysis

**DOI:** 10.3389/fpls.2023.1170004

**Published:** 2023-07-24

**Authors:** Shuai Yuan, Cong Nie, Shangang Jia, Tianqi Liu, Junming Zhao, Jinghan Peng, Weixia Kong, Wei Liu, Wenlong Gou, Xiong Lei, Yi Xiong, Yanli Xiong, Qingqing Yu, Yao Ling, Xiao Ma

**Affiliations:** ^1^ College of Grassland Science and Technology, Sichuan Agricultural University, Chengdu, China; ^2^ Sichuan Academy of Grassland Sciences, Chengdu, China; ^3^ College of Grassland Science and Technology, China Agricultural University, Beijing, China

**Keywords:** *Hordeum*, chloroplast genome, parity rule 2, repeated sequences, hotpot, phylogenic tree

## Abstract

*Hordeum* L. is widely distributed in mountain or plateau of subtropical and warm temperate regions around the world. Three wild perennial *Hordeum* species, including *H. bogdanii*, *H. brevisubulatum*, and *H. violaceum*, have been used as forage and for grassland ecological restoration in high-altitude areas in recent years. To date, the degree of interspecies sequence variation in the three *Hordeum* species within existing gene pools is still not well-defined. Herein, we sequenced and assembled chloroplast (cp) genomes of the three species. The results revealed that the cp genome of *H. bogdanii* showed certain sequence variations compared with the cp genomes of the other two species (*H. brevisubulatum* and *H. violaceum*), and the latter two were characterized by a higher relative affinity. Parity rule 2 plot (PR2) analysis illuminated that most genes of all ten *Hordeum* species were concentrated in nucleotide T and G. Numerous single nucleotide polymorphism (SNP) and insertion/deletion (In/Del) events were detected in the three *Hordeum* species. A series of hotspots regions (*tRNA*-*GGU* ~ *tRNA*-*GCA*, *tRNA*-*UGU* ~ *ndhJ*, *psbE* ~ *rps18*, *ndhF* ~ *tRNA-UAG*, etc.) were identified by mVISTA procedures, and the five highly polymorphic genes (*tRNA-UGC*, *tRNA-UAA*, *tRNA-UUU*, *tRNA-UAC*, and *ndhA*) were proved by the nucleotide diversity (Pi). Although the distribution and existence of cp simple sequence repeats (cpSSRs) were predicted in the three *Hordeum* cp genomes, no rearrangement was found between them. A similar phenomenon has been found in the cp genome of the other seven *Hordeum* species, which has been published so far. In addition, evolutionary relationships were reappraised based on the currently reported cp genome of *Hordeum* L. This study offers a framework for gaining a better understanding of the evolutionary history of *Hordeum* species through the re-examination of their cp genomes, and by identifying highly polymorphic genes and hotspot regions that could provide important insights into the genetic diversity and differentiation of these species.

## 1 Introduction

As secretory organs and active metabolic centers, chloroplasts (cp) are considered the source of energy that drives the evolution of early life (Liu et al., 2018). Although most of the genetic information is provided by the nuclear genome, the cp genome is used to perform variation analysis due to its small size and matrilineal inheritance without gene recombination interference (Gumeni et al., 2017; Shen et al., 2018). Therefore, sequence variation in cp genomes plays a key role in studying plant evolution, and genetic diversity (Xiong et al., 2020a). With the advent of high-throughput sequencing technologies, especially Illumina sequencing, sequence and structure information obtained from the whole cp genome has been elucidated in some vital species (Ogihara et al., 2000; Sajjad et al., 2017). Cp genomes contain several functional genes, such as photosynthesis-related genes, expression-related genes, and biosynthesis-related genes (Bailey et al., 2020). Differential gene detection and phylogeny analysis among genera or families using cp genome sequences is another effective method for studying evolutionary patterns due to the conservative property of cp DNA, mainly in content and arrangement mode. Generally, the structure of the cp genome is quadrantal, containing two inverted repeat (IR) sequences divided by a large single-copy (LSC) region and a small single-copy (SSC) region (Wu et al., 2021). However, four specific *Hordeum* species, *H. pubiflorum*, *H. murinum*, *H. marinum*, and *H. bulbosum*, were a noticeable exception to this typical structure with IR loss or missing introns (Bernhardt et al., 2017). It is noteworthy that this phenomenon was rarely reported in plants in the Poaceae family but it was often found in plants in the Leguminaceae family (Xue et al., 2019).

Derived from the *Triticeae* tribe of the Gramineae family, *Hordeum* L. is composed of approximately 45 species or subspecies, which are distributed in the southern and northern hemispheres, with four species diversity centers, including Southwest Asia, Central Asia, North America, and Southern America (Brassac and Blattner, 2015; and Reinert et al., 2019). The genus *Hordeum* consists of one cultivated species, namely *H. vulgare*, and abundant wild species, such as *H. vulgare* subsp. *spontaneum*, *H. bogdanii*, *H. brevisubulatum*, *H.violaceum* (*H. roshevilzii*), etc. Wild species — which gradually undergo environmental selection — often possess favorable genes such as disease resistance and insect resistance genes and thus are considered important germplasms for genetic improvement (Alyr et al., 2020). Investigation of the genetic diversity and kinship between wild and cultivated species may provide a perspective for the development and utilization of advantageous genes and extension of the genetic basis of cultivars. Previous studies have explored the phylogenetic relationships between wild and cultivated and annual and perennial *Hordeum* species, which mainly depended on the mitochondrial genome sequences (Hisano et al., 2016) or partial nuclear single-copy genome sequence analysis (Jonathan and Blattner, 2015). However, there are relatively few reports on the phylogenetic relationships using complete cp genomes of the genus *Hordeum*. Particularly, large-scale phylogenetic analysis of wild perennial species originating from North Central Asia (*H. bogdanii*, *H. brevisubulatum*, and *H. violaceum*) and those distributed elsewhere is still insufficient. Therefore, performing complete cp genome sequencing of these three wild perennial *Hordeum* species to identify some plastid key genes in interspecific genetic differentiation between the wild and cultivated and/or perennial and annual *Hordeum* species is of great significance, to further improve the phylogenetic relationships and genome structure of the genus *Hordeum*.

Here, complete cp genomes of three wild perennial *Hordeum* species, *H. bogdanii*, *H. brevisubulatum*, and *H. violaceum*, were sequenced and annotated, to determine the cp genome size, nucleotide diversity (Pi), repeat sequences, insertions/deletions (In/Dels), single nucleotide polymorphisms (SNPs). Sequence synteny, relative synonymous codon usage, Parity rule 2 (PR2) analysis, rearrangements, and IR expansions or contractions were evaluated among 10 *Hordeum* species (*H. bogdanii, H. brevisubulatum*, *H. violaceum, H. jubatum*, *H. bulbosum*, *H. marinum*, *H. murinum*, *H. pubiflorum*, *H. vulgare* subsp. *spontaneum*, and *H. vulgare*). In addition, phylogenetic relationships of the sequenced *Hordeum* species from other whole sequenced Poaceae species were revealed. Meanwhile, the degree of variation between wild and cultivated and annual and perennial *Hordeum* species was further evaluated. This study contributes to the expansion of the cp genome database.

## 2 Methods

### 2.1 Plant material, DNA extraction and sequencing

Three *Hordeum* species, *H. bogdanii, H. brevisubulatum*, and *H. violaceum*, were from NPGS (National Plant Germplasm System of the United States; **Supplementary Table 1**). In total, 100 mg leaves were harvested at the three-leaf stage, and then total genomic DNA was extracted using the plant DNA Extraction Kit (Tiangen, Beijing, China) as per manufacture’s instruction. DNA concentration was quantified using 0.1% agarose gel, libraries were established, and DNA with good quality was selected and sequenced using the Illumina NovaSeq platform with a read length of PE150.

### 2.2 Chloroplast genome assembly and annotation

The complete circular genome sequence cannot be directly obtained by one-time splicing because of the characteristics of next-generation sequencing (NGS), genomic repeats, a specific structure of the genome, and related factors. Therefore, a different complicated strategy was performed: The kernel modules were assembled using the SPAdes v3.10.1 (Saint Petersburg State University, Saint, Russia) (Safonova et al., 2014) software for the cp genome of three species, which is not dependent on the reference genome. The contig was obtained using the kmer iterative extend seed. The SSPACE v2.0 procedure was used (BaseClear BV, Einsteinweg, Leiden, The Netherlands) (Boetzer et al., 2011) to acquire scaffolds by connecting contig sequences. The gap of scaffolds sequence was constructed using Gapfiller V2.1.1 procedure (BaseClear BV, Einsteinweg, Leiden, The Netherlands) to assemble a complete pseudo sequence (Boetzer and Pirovano, 2012). The alignment-correction method was used to align the sequencing sequence into the pseudo genome, which was later rearranged according to the cp structure of the three species, thereby obtaining a complete cp circular genome sequence.

Cp gene structure annotation plays an important role in cp genome sequencing. Blast v2.2.25 (U.S. National Library of Medicine 8600 Rockville Pike, Bethesda MD, 20894 USA) (Kent and Brumbaugh, 2002) was used to align CDS sequences of cp genome in NCBI. The gene annotation results of cp genomes for three *Hordeum* species were acquired using a manual correction. Moreover, to obtain gene annotation, rRNA and tRNA sequences of cp genomes were aligned in NCBI (https://www.ncbi.nlm.nih.gov/) database using HMMER v3.1b2 (HHMI/Harvard University, Boston, USA; The European Bioinformatics Institute, Cambridge, UK) (Finn et al., 2011) and Aragorn v1.2.38 programs (Murdoch University, Western Australia, Australia; Lund University, Lund, Sweden) (Dean and Bjorn, 2004). In addition, *H. vulgare* subsp. *spontaneum* (KC912688.1) was used as a reference sequence for quality control of the cp genome after assembly.

### 2.3 Prediction of repetitive sequences

The Simple Sequence Repeats (SSRs) markers are a class of tandem repeats with motifs consisting of several nucleotides group (usually 1~6) as repeating units. The SSR marker is called cpSSR marker on cp genomes. CpSSR were identified and analyzed using the software MISA v1.0 (Leibniz Institute of Plant Genetics and Crop Plant Research (IPK) Gatersleben, Corrensstr. 3, 06466 Seeland, Germany) (Beier et al., 2017). CpSSR parameters were described as A-B, with A representing the number of repetitions and B representing the total number of the base unit in a sequence. For example, 1-8 indicates more than 8 repetitions of a single-base, 2-5 indicates more than 5 repetitions of a double-base, 3-3 more than three repetitions of triple-base, 4-3, 5-3, 6-3 and so on. Furthermore, the interspersed repeats sequences, which are a different kind of repetitive sequences from tandem repeats and have both forward and palindromic repeats (including reverse and complementary) with a minimum size of 15 bp, sequence coherence of more than 90% and are distributed throughout the genome, were identified using the Vmatch v2.3.0 (http://www.vmatch.de/) program.

### 2.4 Relative synonymous codon usage and parity rule 2 analysis

The degeneracy of codons show that each amino acid has one to six codons. The heterogeneity of synonymous codon usage is called Relative Synonymous Codon Usage (RSCU). To highlight the relative biasness between amino acids and codons, the RSCU was analyzed using the MEGA v10.1.8 program (Kumar et al., 2008).

The complete cp genomes of the three *Hordeum* species sequenced in this study and seven other *Hordeum* species (*H. bulbosum*, *H. jubatum*, *H. marinum*, *H. murinum*, *H. pubiflorum*, *H. vulgare* subsp. *spontaneum*, *H. vulgare*) were downloaded from the NCBI database and used for PR2 analysis to evaluate nucleotide usage bias in the coding genes of them (Wei et al., 2014). Base A, T, C and G content at the third site of synonymous codons were calculated using the MEGA v10.1.8 software.

### 2.5 Analysis of sequences variation and Ka/Ks

SNP (Single Nucleotide Polymorphism) refers to the DNA sequence polymorphism caused by the variation (insertions or deletions (In/Dels)) of a single nucleotide at the genomic level and accounts for more than 90% of known polymorphisms. The cp genomes of three *Hordeum* materials were aligned using MAFFT program, version v7.310 (https://mafft. cbrc. jp/alignment/software/) (Standley, 2013) to identify SNP and In/Dels. In addition, the nucleotide diversity (Pi) and Ka/Ks in this study were calculated using the conjunct genes and protein-coding genes of the three *Hordeum* materials detected. Base mutation, including non-Synonymous mutations (Ka) and synonymous mutations (Ks) causes changes in amino acids, which ratios > 1 is called a positive selection effect and < 1 is named a purified selection effect. Pi is considered an important tool that able to reveal the variation of size of nucleic acid sequences, and a range of potential molecular markers can be provided based on the regions of high variability for population genetics (Meng et al., 2018). The Ka/Ks and Pi values were calculated using KaKs_Calculator v2.0 (https://sourceforge.net/projects/kakscalculator2/) (Zhang et al., 2006) and VCFTOOLS (Danecek et al., 2011), respectively. Nevertheless, before achieving the above tasks, the CDS sequences of the conjunct genes in each species were globally aligned using MAFFT software.

### 2.6 Multiple Cp genomes alignment

Alignment and collinearity of 10 *Hordeum* species complete cp genomes, *H. bogdanii, H. brevisubulatum*, *H. violaceum, H. jubatum*, *H. bulbosum*, *H. marinum*, *H. murinum*, *H. pubiflorum*, *H. vulgare* subsp. *spontaneum*, and *H. vulgare*, was analyzed using Mauve (Darling et al., 2004) and Mvista tools (http://genome.lbl.gov/vista/mvista/submit.html). The IRSCOPE online software (https://irscope.shinyapps.io/irapp/) was used to evaluate the expansion or contraction of IR and SC regions boundary for six species (*H. bogdanii*, *H. brevisubulatum*, *H. violaceum*, *H. jubatum*, *H. vulgare* subsp. *spontaneum*, and *H. vulgare*).

### 2.7 Phylogenetic analysis

A total of 28 Poaceae species published in NCBI (**Supplementary Table 2**), and three *hordeum* species (*H. bogdanii* (CNS0491101), *H. brevisubulatum* (CNS0491102), *H. violaceum* (CNS0491103)) that in this study were sequenced to establish the phylogenetic tree. *Saccharum spontaneum* (LN896360.1) and *Sorghum bicolor* (NC008602.1) were the outgroups. MAFFT and RAxML v8.2.10 software (https://cme. h-its. org/exelixis/software. html) that follow GTR model and Hill Climbing algorithm were carried out to achieve the multi-sequence alignment and construction of the phylogenetic tree for different species, respectively.

## 3 Results

### 3.1 Characteristics of Cp genomes of six *Hordeum* species

**Figure 1:**
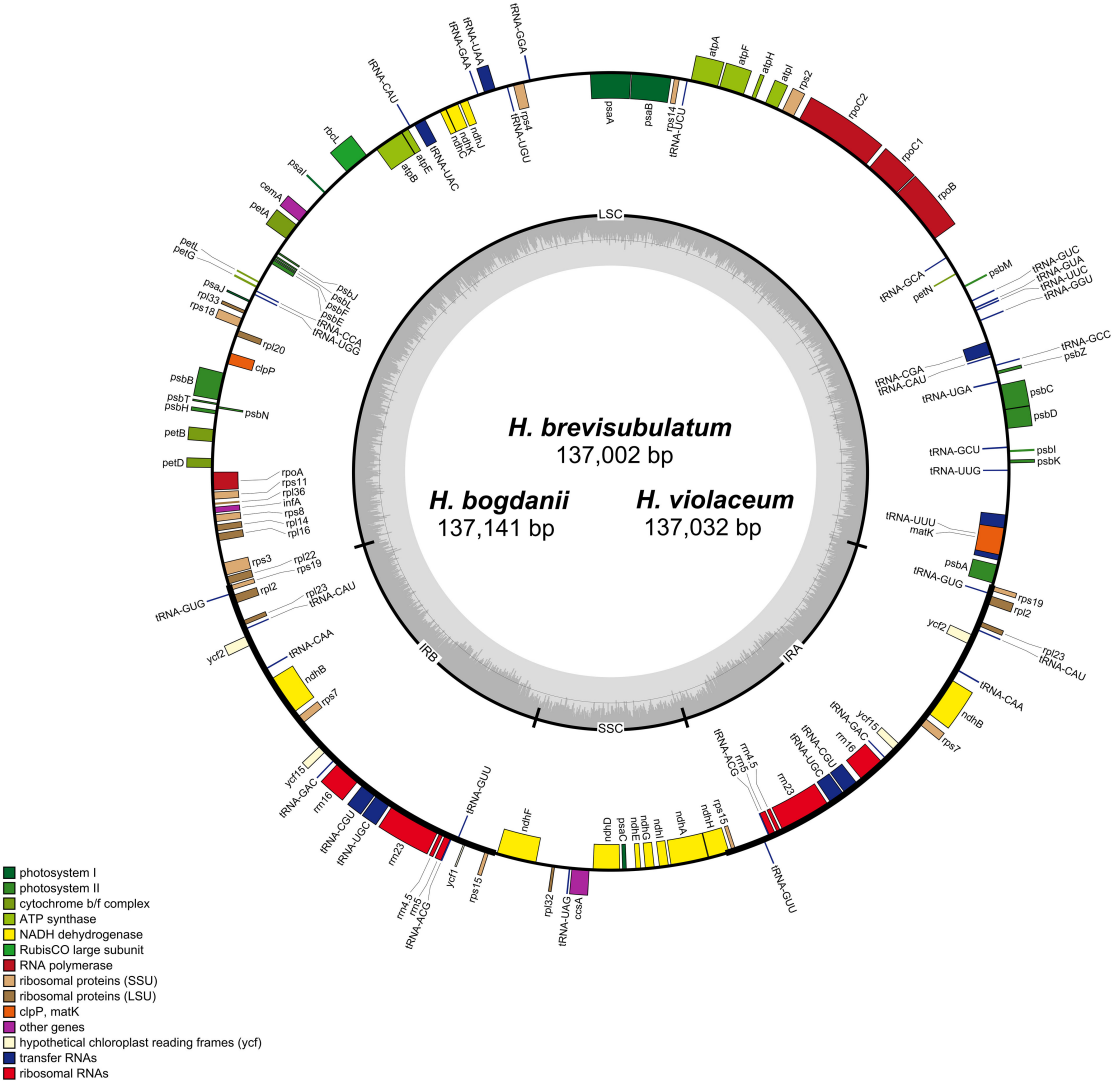
Gene maps of *H. bogdanii*, *H. brevisubulatum, and H. violaceum* cp genomes. Genes inside and outside the circle undergo clockwise and counterclockwise transcription in the gene map. Dark gray and light gray color represent guanine and cytosine (GC) content and adenine and thymine (AT) content, respectively.

Due to the loss of the IR region in the cp genomes of *H. pubiflorum*, *H. murinum*, *H. marinum*, and *H. bulbosum*, cp genome characteristics of only six *Hordeum* species, *H. bogdanii*, *H. brevisubulatum*, *H. violaceum*, *H. jubatum*, *H. vulgare* subsp. *spontaneum*, and *H. vulgare* were selected for comparison of cp genome characteristics (**Figure 1**). This comparison also included IR expansion and contraction. *H. vulgare* had the smallest cp genome size (136,462 bp) compared with that of the other five species (*H. bogdanii* (137,141 bp), *H. brevisubulatum* (137,002 bp), *H. violaceum* (137,032 bp), and *H. spontaneum* (136,536 bp), *H. jubatum* (136,826 bp), while it also had the highest GC content and total number of genes. Illumina paired-end sequencing yielded 26,262,890, 25,330,242, and 25,890,515 ReadSum (pair-end reads) from *H. bogdanii, H. brevisubulatum*, and *H. violaceum*, respectively. Q20 and Q30 (the percentage of bases with a mass value ≥20 and ≥30, respectively) were both more than 85%. The three perennial species (*H. bogdanii, H. brevisubulatum*, and *H. violaceum*) belonged to a typical quadrantal model, consisted of two copies of IR regions (IRs 21,573-21,587 bp), and were separated by LSC (81,128-81,169 bp) and SSC (12,728-12,798 bp) regions, which are the common feature of the majority of plants in the Poaceae family (**Figure 1**, **Table 1**). The overall GC content in the cp genomes of *H. bogdanii, H. brevisubulatum*, and *H. violaceum* was 38.23, 28.28, and 38.27%, respectively, and the percentage distributed in the IR regions was the highest than that in LSC and SSC regions. A total of 129, 131, and 131 genes were located in the complete cp genomes of *H. bogdanii, H. brevisubulatum*, and *H. violaceum*, respectively. Thirty-eight ribosomal RNA (rRNA) genes, 8 transfer RNA (tRNA) genes, and 85 messenger RNA (mRNA) genes were distributed in both *H. brevisubulatum* and *H. violaceum*. Interestingly, the annual cultivated species (*H. vulgare*) had the largest number of genes compared with the other five species, but these genes these genes were all attributed to tRNA.

**Table 1:** Comparison of the six *Hordeum* chloroplast genomes.

Species	Improvement status	Size (bp)				GC content (%)				tRNA	rRNA	mRNA	Genes
		Cp genome	LSC	SSC	IR	Cp genome	LSC	SSC	IR				
*H. bogdanii*	Wild, perennial	137141	81169	12798	21587	38.23	36.20	32.10	43.87	38	8	83	129
*H. brevisubulatum*	Wild, perennial	137002	81128	12728	21573	38.28	36.25	32.27	43.87	38	8	85	131
*H. violaceum*	Wild, perennial	137032	81155	12731	21573	38.27	36.24	32.26	43.87	38	8	85	131
*H. jubatum*	Wild, perennial	136826	80901	12665	21630	38.24	36.19	32.32	43.81	39	8	82	129
*H. vulgare* subsp. *spontaneum*	Wild, annual	136536	80612	12778	21573	38.30	36.30	32.25	43.84	39	8	83	130
*H. vulgare*	Cultivate, annual	136462	81671	12701	21045	38.32	36.31	32.33	43.83	48	8	83	139

Out of the 113 genes were shared by the five cp *Hordeum* genomes (*H. bogdanii, H. brevisubulatum*, *H. violaceum*, *H. vulgare* subsp. *spontaneum*, and *H. vulgare*) (**Table 2**). 46 were annotated to photosynthesis-related genes such as the large subunit of rubisco, a subunit of photosystem I, a subunit of photosystem II, a subunit of ATP synthase, cytochrome b/f complex, c-type cytochrome synthesis, and subunit of NADH dehydrogenase. Thirty-four genes were involved in self-replication, of which 30 genes and 4 genes were related to tRNA and rRNA, respectively. In addition, 12 genes encoding ribosomal proteins, as well as 14 genes were assembled into transcription. Interestingly, *trnI-GAU*, *trnG-UCC*, *rps12*, and *rps16* genes were unique to two annual species (*H. vulgare* subsp. *spontaneum* and *H. vulgare*), while *trnT-CGU* and *trnS-CGA* genes were specific to three perennial species (*H. bogdanii, H. brevisubulatum*, and *H. violaceum*). More mutations may accumulate in introns because they are less constrained by natural selection than exons (Xiong et al., 2020b). Ten genes that contained a single intron in three cp genomes were collected (**Supplementary Table 3**).

**Table 2:** List of genes annotated in the plastomes of the three wild perennial *Hordeum* species (*H. bogdanii, H. brevisubulatum*, and *H. violaceum*) from Central Asia and two annual species (*H. vulgare* subsp. *spontaneum* and *H. vulgare*).

Category	Function	Name of gene							
Self-replication (34)	Ribosomal RNA genes	**rrn4.5**	**rrn5**	**rrn16**	**rrn23**				
	Transfer RNA genes	**trnR-ACG**	**trnL-CAA**	**trnV-GAC**	**trnH-GUG**	**trnN-GUU**	trnA-UGC^*^	trnT-CGU^*/bbv^	trnS-CGA^*/bbv^
		**trnM-CAU**	**trnI-GAU** ^*/^ **^vul^**	trnG-UCC^*/vul^	trnK-UUU^*^	trnL-UAA^*^	trnV-UAC^*^	trnC-GCA	trnG-GCC
		trnS-GCU	trnS-GGA	trnT-GGU	trnY-GUA	trnD-GUC	trnL-UAG	trnR-UCU	trnS-UGA
		trnP-UGG	trnT-UGU	trnE-UUC	trnQ-UUG	trnF-GAA	trnW-CCA		
Ribosomal proteins (12)	Small subunit of ribosome (SSU)	rps2	rps3	rps4	**rps7**	rps8	rps11	**rps12^vul^**	rps14
		**rps15**	rps16^*/vul^	rps18	**rps19**				
Transcription (14)	Large subunit of ribosome (LSU)	rpl2^*^	rpl14	rpl16	rpl20	rpl22	**rpl23**	rpl32	rpl33
		rpl36							
	RNA polymerase subunits	rpoA	rpoB	rpoC1	rpoC2				
	Translation initiation factor	infA							
Photosynthesis related genes (46)	RubisCO large subunit	rbcL							
	Subunits of photosystem I	psaA	psaB	psaC	psaI	psaJ			
	Subunits of photosystem II	psbA	psbB	psbC	psbD	psbE	psbF	psbH	psbI
		psbJ	psbK	psbL	psbM	psbN	psbT	psbZ	
	Subunits of ATP synthase	atpA	atpB	atpE	atpF^*^	atpH	atpI		
	Cytochrome b/f complex	petA	petB	petD	petG	petL	petN		
	C-type cytochrome synthesis gene	ccsA							
	Subunits of NADH dehydrogenase	ndhA^*^	ndhB^*^	ndhC	ndhD	ndhE	ndhF	ndhG	ndhH
		ndhI	ndhJ	ndhK					
Other genes (6)	Maturase	matK							
	Protease	clpP							
	Chloroplast envelope membrane protein	cemA							
	Hypothetical open reading frames	ycf1	**ycf2**	ycf3^#/bvv^	ycf4^bvv^				
Unknown function (1)	Plant protein of unknown function	**ycf15^bbb^**							

*, gene containing a single intron; #, gene containing two introns; Genes in bold correspond to genes that are located in the IRs and hence are duplicated; bbv, genes that are particular for *H. bogdanii, H. brevisubulatum* and *H. violaceum*; vul, genes that are particular for *H. vulgare* subsp. spontaneum, and *H. vulgare*; bvv, genes that are particular for *H. brevisubulatum*, *H. violaceum*, *H. vulgare* subsp. *spontaneum*, and *H. vulgare*.

### 3.2 Repeat sequence analysis

**Figure 2:**
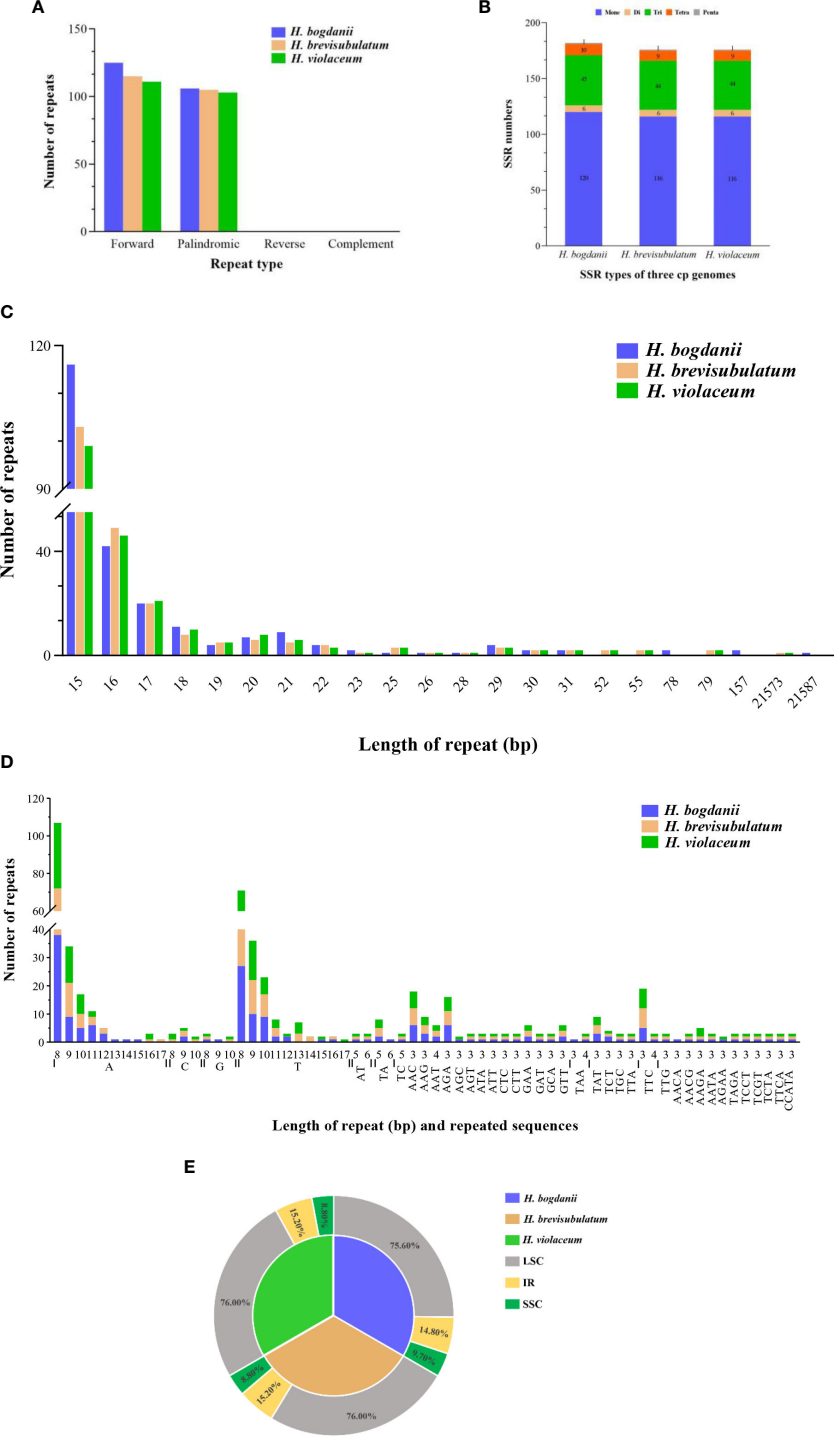
Simple sequence repeats (SSRs) and scattered repetitive sequences in the three *Hordeum* cp genomes. **(A)** frequency of repeat types; **(B)** compare of the number of SSR type in the three *Hordeum* cp genomes; **(C)** frequency of repeats length; **(D)** motifs in the cp genome of *Hordeum*; **(E)** Distribution region of repeating sequences of three *Hordeum* cp genome. IR, inverted repeat; LSC, large single-copy; SSC, small single-copy.

Two different types of repeat sequences, which includes scattered repetitive sequences (palindrome repeats and direct repeats) and simple sequence repeats (SSR), were carefully analyzed Using MISA v1.0 and Vmatch v2.3.0, respectively. A total of 231 (forward type, 125 and palindromic type, 106), 220 (forward type 115 and palindromic type, 105), and 218 (forward type,115 and palindromic type 103) scattered repetitive sequences were predicted in *H. bogdanii, H. brevisubulatum*, and *H. violaceum*, respectively (**Figure 2A**). Their common characteristic is the number of repeats reached the peak at a repeat length of 15 bp (**Figure 2C**). SSR, a tandem repeat sequence of dozens of nucleotides generally composed of a series of repeat units (1-6 bp in length), was distributed throughout the genome. A total of 182 SSR in the cp genome of *H. bogdanii* was detected, which was greater than that of *H. brevisubulatum* (178) and *H*. *violaceum* (176) (**Figure 2B**). The number of mononucleotides (primarily poly-A or poly-T) accounted for the largest proportion of total SSR, which was above 59% (**Figure 2B**). Interestingly, trinucleotide (AGC) and tetranucleotide (AACA and AGAA) SSR were found only in *H. bogdanii*, and other types of SSR nucleotides in the cp genome of the three wild perennial *Hordeum* species were predicted with a fixed distribution (**Figure 2D**), which warrants further investigation in the future. The mononucleotide T was repeated 13 times and was unique to *H. brevisubulatum* and *H. violaceum* (**Figure 2D**). Furthermore, the majority of SSR were distributed in the LSC region, of which the proportion of *H. bogdanii* was 75.6%, slightly lower than that of *H. brevisubulatum* (76%) and *H*. *violaceum* (76%) (**Figure 2E**).

### 3.3 Relative synonymous codon usage and PR2-plot analysis

**Figure 3:**
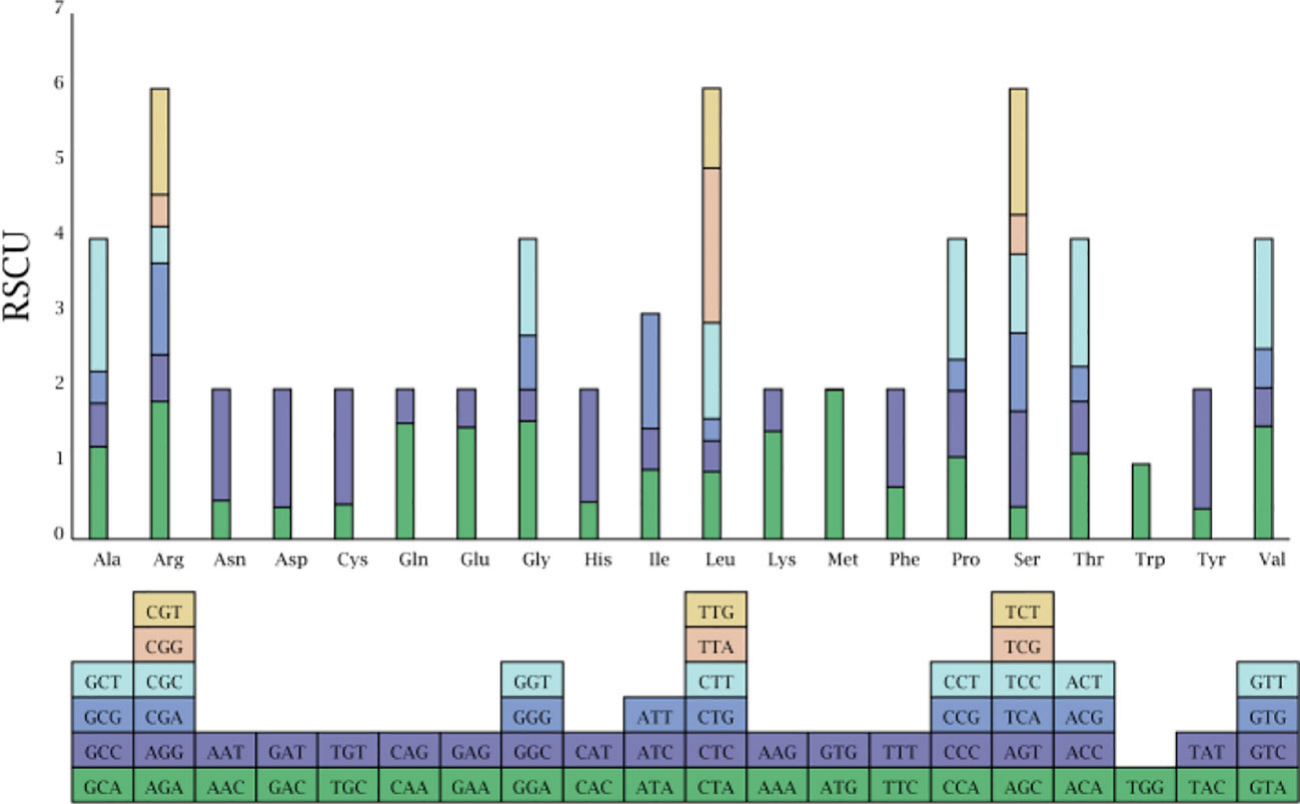
Relative frequency of synonymous codon for the twenty amino acids in the three *Hordeum* species chloroplast genomes.

RSCU, which is caused by the unequal usage of a synonymous codon, was further analyzed (**Figure 3**). Each amino acid corresponds to at least one codon and at most six codons owing to the redundancy of codons. RSCU values for the initial codon (AUG) were 1.987, 1.983, and 1.987 in *H. bogdanii*, *H. brevisubulatum*, and *H*. *violaceum*, respectively. RSCU values for termination codons, UAA, UAG, and UGA, were 1.771, 0.651, and 0.578 in *H. bogdanii*, 1.730, 0.671, and 0.600 in *H. brevisubulatum*, and 1.730, 0.671, and 0.600 in *H*. *violaceum*, respectively. Codons with RSCU values >1, which are usually considered to be preferred codons, accounted for 51.61% (32/62) of codons, and the third nucleotide of most codons was biased towards either A or U. Notably, only one codon, UGG (corresponding to tryptophan), showed no bias in the three *Hordeum* species, and its RSCU was 1.00.

**Figure 4:**
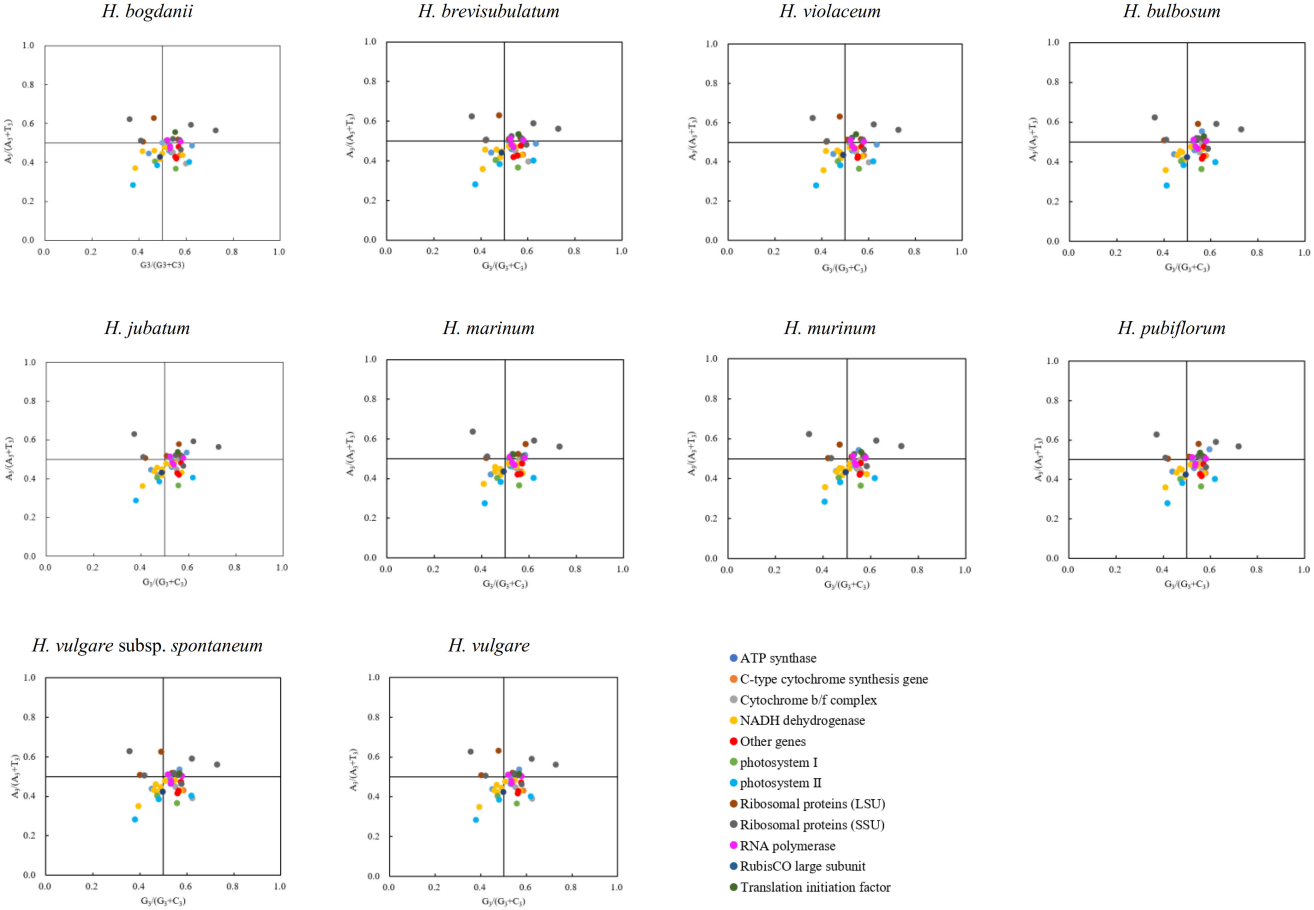
PR2-plot analysis of cp genomes ten *Hordeum* species. Base A, T, C and G content at the third site of synonymous codons were replaced through A_3_, T_3_, C_3_ and G_3_, respectively.

Forty-four coding sequences (CDS, ≥300 bp long) containing start (ATG) and stop (TAG, TGA, TAA) codons were collected from the 10 cp genomes, to carry out PR2-plot analysis to further understand codon bias (**Figure 4**). The results showed that the 44 genes of the 10 species were not evenly distributed within the four regions, but mainly in G_3_/(G_3_+C_3_) > 0.5 and A_3_/(A_3_+T_3_) < 0.5 regions. This suggests that there may be a bias towards G and T bases at the third position of synonymous codons, which needs further investigation.

### 3.4 In/Dels and SNPs

**Figure 5:**
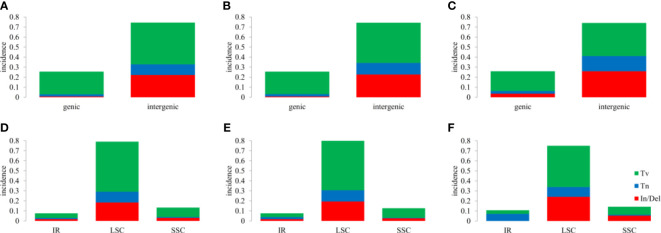
Overview of single nucleotide polymorphisms (SNPs) and Insertions/Deletions (In/Dels). **(A, B)**, **(C, D)**, and **(E, F)** the differences between *H. bogdanii* vs *H. brevisubulatum*, *H. bogdanii* vs *H. violaceum* and *Hordeum brevisubulatum* vs *Hordeum. violaceum*. Tv, transversion; Tn, transition; In/Del, insertion/deletion; IR, inverted repeat; LSC, large single-copy; SSC, small single-copy.

InDels and SNPs (mainly containing Tn (transition) and Tv (transversion)) were detected among the three *Hordeum* cp genomes using MAFFT software (Standley, 2013). A total of 109, 112, and 33 In/Dels were identified in *H. bogdanii* vs *H. brevisubulatum*, *H. bogdanii* vs *H. violaceum*, and *H. brevisubulatum* vs. *H. violaceum*, respectively, in which 4 InDels were discovered in the coding sequence (**Supplementary Table 4**). There were similar quantities of Tn and Tv in both *H. bogdanii* vs *H. brevisubulatum* (Tn = 61, Tv = 304) and *H. bogdanii* vs *H. violaceum* (Tn = 66, Tv = 298), most of which were encoded in the noncoding sequence. However, 19 Tn (2 coding, 17 noncoding) and 60 Tv (23 coding, 37 noncoding) were detected during *H*. *brevisubulatum* vs *H. violaceum*. Interestingly, we found that both InDels and SNPs were mainly concentrated in LSC and the intergenic region for each pairwise comparison, while InDels did not occurred in the IR region of *H*. *brevisubulatum* vs *H. violaceum* (**Figure 5**).

**Figure 6:**
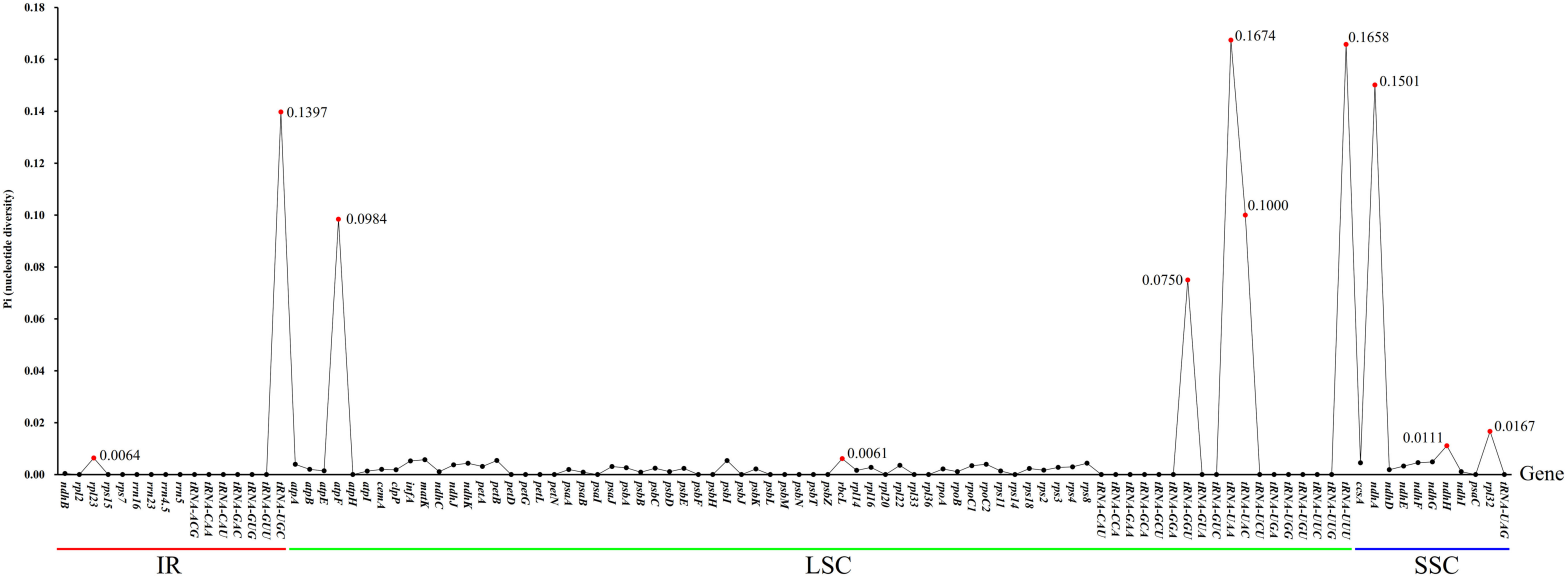
The nucleotide diversity (Pi) calculated by 101 genes shared in three wild perennial *Hordeum* species. Genes with Ka/Ks value > 1 are highlighted in red; The genes above the red line, green line and blue line were located in IR, LSC and SSR regions, respectively.

The non-synonymous/Synonymous mutation ratio (Ka/Ks) ratio of 83 common protein-coding genes in cp genomes of the three *Hordeum* species was calculated using Ka/Ks Calculator software (Zhang et al., 2006) (**Supplementary Table 3**). Ka/Ks values of *H. bogdanii* vs *H. brevisubulatum*, *H. bogdanii* vs *H. violaceum*, and *H. brevisubulatum* vs *H. violaceum* were 16, 19, and 2, respectively. In addition, the Ka/Ks values of some genes (*ropB*, *atpI*, *psaB*, etc.) could not be computed because Ka or/and Ka was 0, which suggests that these genes were relatively conservative without any Ka or Ks nucleotide substitution. Pi values were calculated using VCFTOOLS software. A total of 101 common genes in the three wild perennial *Hordeum* species were examined, whose Pi values ranged between 0 to 0.1674 (**Figure 6**). It is noteworthy that relatively higher Pi values (Pi ≥ 0.1) were detected in five genes, including *tRNA-UGC*, *tRNA-UAA*, *tRNA-UUU*, *tRNA-UAC*, and *ndhA*. Meanwhile, these genes were also among those with Ka/Ks > 1. Moreover, other genes with a Pi ≥ 0.1 were found in single-copy (SC) rather than IR regions, except for *tRNA-UGC*.

### 3.5 Whole Cp genomes comparison with ten *Hordeum* species

**Figure 7:**
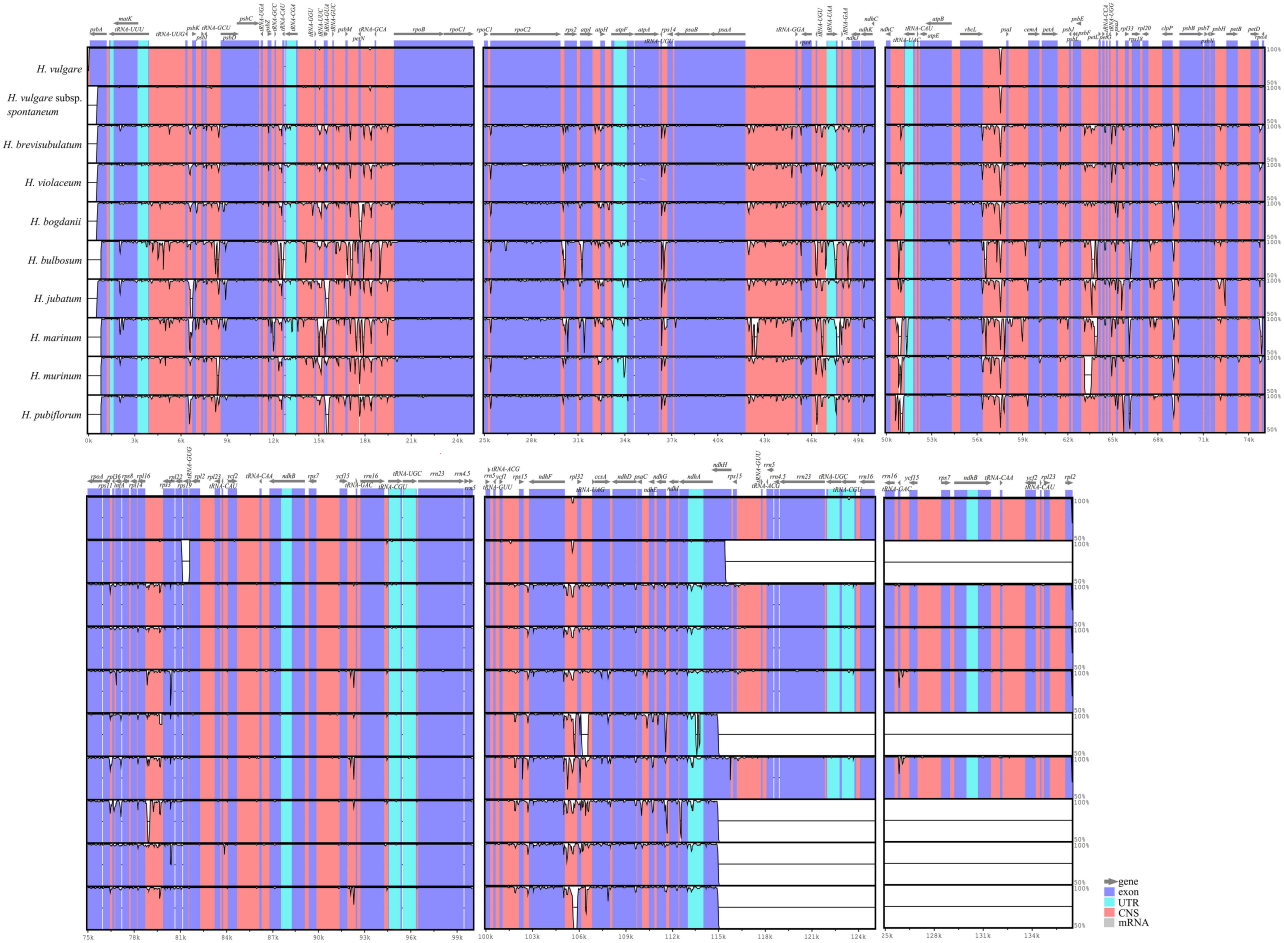
Alignment of the ten *Hordeum* species cp genome sequences. Exon, untranslated region (UTR), conserved noncoding sequences (CNS), and mRNA were marked by different colors. The x-axis and level a clinic columnar strip express the paratactic and sequences stability in the cp genome and the peaks represent hotspot regions.

To understand the sequence divergence between wild and cultivated, as well as annual and perennial species in genus *Hordeum*, and elaborate further on the evolutionary events that occurred, including gene mutation, rearrangement and loss, we analyzed and compared the cp genomes of two annual species (one cultivated species, *H. vulgare* and one wild species, *H. vulgare* subsp. *spontaneum*), and eight perennial wild species (*H. bogdanii, H. brevisubulatum, H. violaceum, H. bulbosum, H. jubatum, H. marinum, H. murinum*, and *H. pubiflorum*) were compared and analyzed. It was found that the coding region is more conservative than the non-coding region, as well as the divergence frequency was higher in the LSC and SSC region than in IR region (**Figure 7**). The two annual species (especially *H. vulgare*) had many conserved regions compared with the other eight wild perennial species, this was the case in the CNS (Conserved Noncoding Sequences) of LSC and SSC regions. The highly variable regions are called hotspots regions, and these regions were mainly concentrated in small RNA molecules such as *tRNA*-*GGU* ~ *tRNA*-*GCA*, *tRNA*-*UGU* ~ *ndhJ*, *psbE* ~ *rps18*, *ndhF* ~ *tRNA-UAG*. Furthermore, MAUVE software revealed rearrangement events with scanty genes in the cp genomes of 10 species (**Supplementary Figure 1**).

### 3.6 IR expansion and contraction

**Figure 8:**
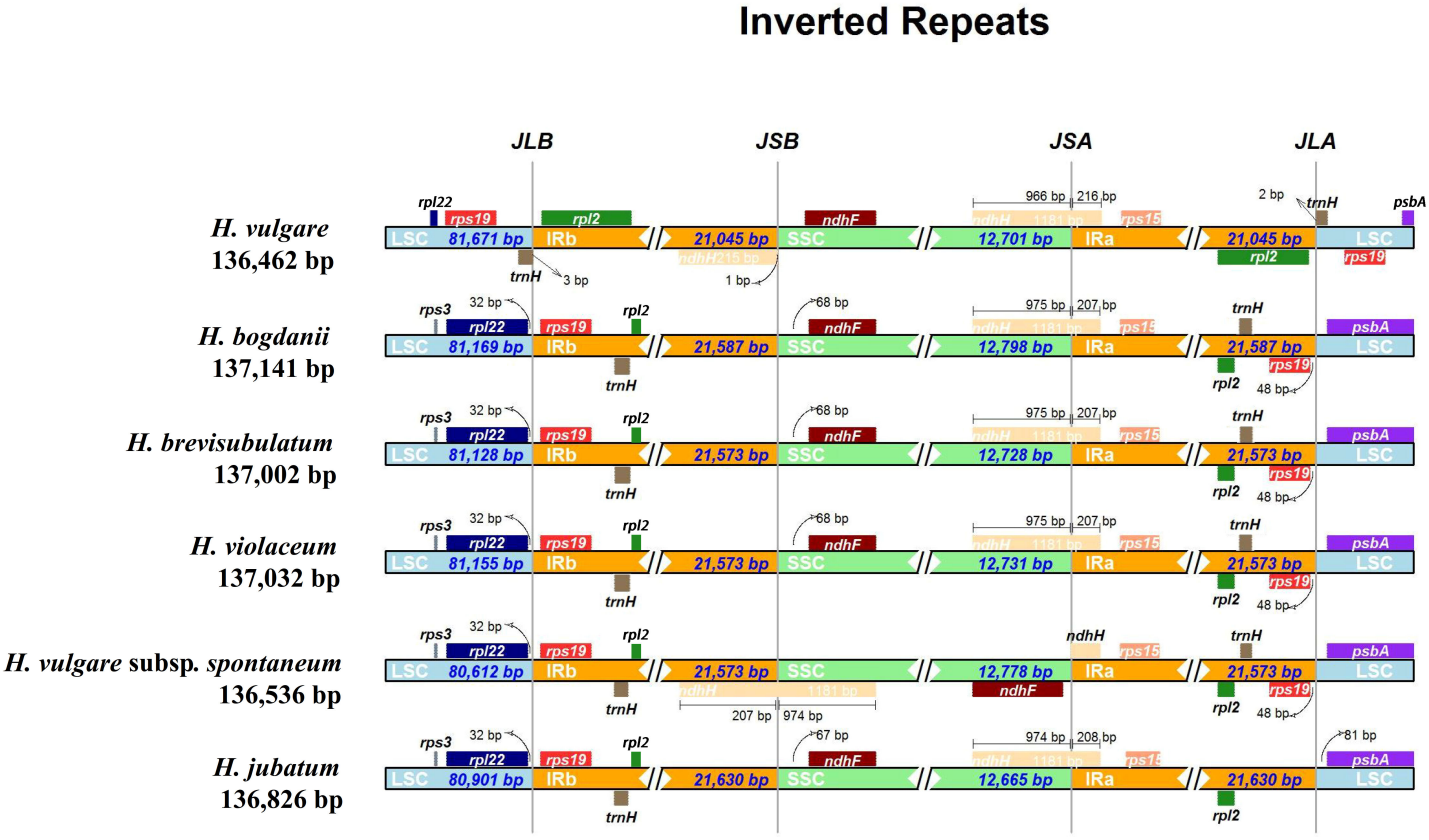
IRscope analysis of the six *Hordeum* cp genomes. JLB, JSB, JSA, and JLA represent the junction of LCS and IRb, SSC and IRb, SSC and IRa, and LSC and IRa region, respectively.

Expansion and contraction of IR regions, recognized as an evolutionary event, are generally concentrated in the junction of IR/SSC or IR/LSC. Moreover, this phenomenon is the primary cause of the variation of cp genomes size. Therefore, the IR borders of six species in the *Hordeum* genus were compared to explore their differences. The species studied included two annuals (including one cultivated species, *H. vulgare* and one wild species, *H. vulgare* subsp. *spontaneum*), and four perennial wild species (*H. bogdanii, H. brevisubulatum, H. violaceum*, and *H. jubatum*) (**Figure 8**). The results showed significant differences in the junction sites between the annual and perennial species. The genes *ndhF*-*ndhH* and *rpl2*-*trnH*-*psbA*-*rpl22*-*rps19* were found close in SSC/IR and LSC/IR boundaries, respectively. The *ndhH* genes of the other five species ranged from 207 (*H. bogdanii*, *H. brevisubulatum*, *H. violaceum*) to 216 (*H. vulgare*) bp in IRa region throughout the SSC/IRa junction, with the exception of *H. vulgare* subsp. *spontaneum*. Two genes, *trnH* and *rpl2*, were found near the junction of LSC/IR region in *H. vulgare*, whereas the genes around this junction region of the other five species were *rpl22* and *rps19* genes. Additionally, we observed that only *e ndhH* gene for *H. vulgar* was separated from SSC/IRb boundary with 1 bp.

### 3.7 Phylogenetic relationships

**Figure 9:**
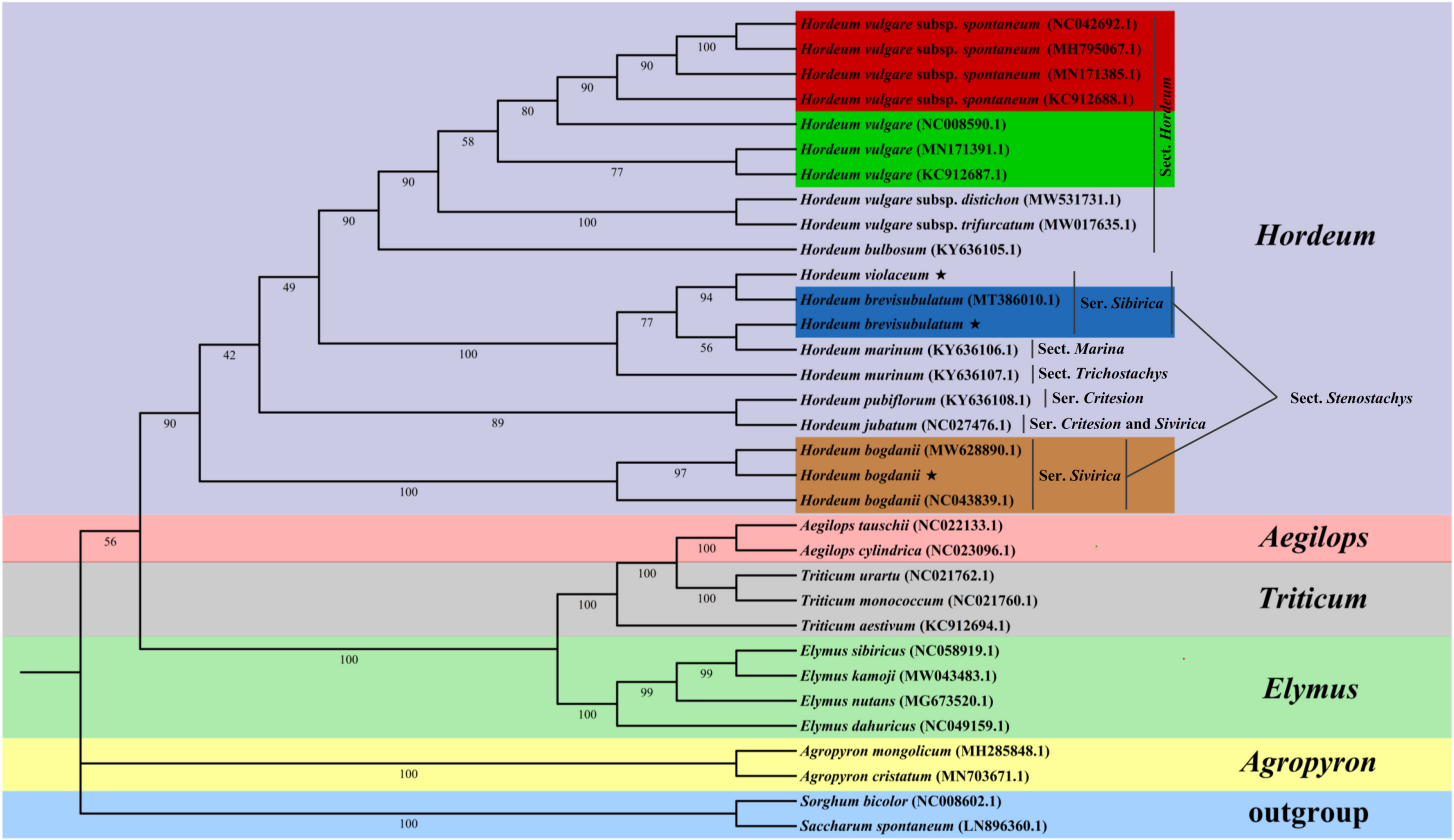
ML phylogenetic tree of 31 Poaceae species, with *Saccharum spontaneum* and *Sorghum bicolor* as outgroups. The bootstrap values are shown at the nodes; *H. vulgare* subsp. *spontaneum*, *H. vulgare*, *H. brevisubulatum*, and *H. bogdanii* species of different accession were represented by the base color of red, green, blue, and orange, respectively.

The phylogenetic position of Triticeae was identified based on the cp genomes sequences of three studied *Hordeum* species and other 28 species downloaded from NCBI (**Figure 9**). The structure of this phylogenetic tree of these species conformed with the classical botanical classification. Twelve *Hordeum* species were divided into six sub-groups, among which *H. brevisubulatum*, and *H. violaceum* were in the same sub-groups, and *H. bogdanii* is further distant from them. Different accessions of the same species are placed in the same subgroup. In addition, genus *Hordeum* was more closely related to the species of *Elymus*, *Aegilops*, *Triticum* than to *Agropyron*.

## 4 Discussion

### 4.1 Characteristics of Cp genomes of *Hordeum* species

The total size and GC content of cp genomes were not significantly different among the three wild perennial *Hordeum* species (*H. bogdanii*, *H. brevisubulatum*, and *H. violaceum*). These results revealed that the cp genome size and GC content of Poaceae are highly conserved, and the occurrence of variation may help us to better understand the unique variation among species or subspecies (Liu et al., 2019). A total of 129, 131, and 131 genes were detected in the cp genomes of *H. bogdanii*, *H. brevisubulatum*, and *H. violaceum*, respectively. Notably, two mRNA genes, *ycf3* and *ycf4*, which were detected in these transformants and have been shown to contribute to the unstable accumulation of photosystem I complexes in the thylakoid membranes (Boudreau et al., 1997), were not found in *H. bogdanii*. This may be because two genes were transferred from the cp genome of *H. bogdanii* to its nuclear genome during the evolution of the species (Xiong et al., 2020). Two transfer RNA genes (*trnG-UUC* and *trnI-GAU*) and two small subunit of ribosome genes (*rps12* and *rps16*) were found to be unique to only two annual *Hordeum* species, including one wild species (*H. vulgare* subsp. *spontaneum*) and one cultivated species (*H. vulgare*). However, the functions of these four genes require further validation in the future. Genes specific for cultivated species (*H. vulgare*) in this study were not identified. This may be due to genetic changes may not exist in the cp genome but rather in the nuclear genome during plants domestication. Typically, cp genomes of Poaceae species are highly conserved in structure, which is a typical quadripartite (the IR region is separated by LSC and SSC). However, in some plants, cp genomes contain only one IR region (alfalfa) (Tao et al., 2016) or lack the IR region (algae) (Xue et al., 2019). *H. bulbosum*, *H. marinum*, *H. murinum*, and *H. pubiflorum* also fall into this category, with linear cp genomes without the IR region (Bernhardt et al., 2017). Therefore, the cp genome characteristics of these four *Hordeum* species were not analyzed and compared in the current study. However, cp genome characteristics of only two annual species (*H. vulgare*, and *H. vulgare* subsp. *spontaneum*) and four perennial species (*H. bogdanii*, *H. brevisubulatum*, *H. violaceum*, and *H. jubatum*) were analyzed and compared. The result demonstrated that the size and GC content of cp genomes of the six *Hordeum* species ranged from 136,462 to 137,141 bp and 38.23% to 38.32%, respectively, indicating that the cp genome length and GC content of synanthropic species were not significantly different, while the number of genes (139) in cultivated species were more abundant compared with that in wild species. The reason may be that natural selection has led to an accelerated rate of gene loss in wild species (Vishwakarma et al., 2017). It is well known that gene degradation and even loss occur because the cp genome of angiosperms evolves relatively fast (Lei et al., 2016). Our study found no significant difference in the total number of genes among the five wild *Hordeum* species, which ranged from 129 to 131 (**Table 1**), which was significantly lower than that of *H. vulgare* (139), with a maximum gap of 10 genes and a minimum of 8 genes, such as *rps12*, *rps16*, etc. There is evidence that these genes have been lost in *Ulmus* (Zuo et al., 2017) and *Orchidaceae* (Jing et al., 2014).

Introns, which are located in the non-coding region, typically have higher mutation rates than exons, as their functions are often more restricted (Gan et al., 2018). Nevertheless, it is noteworthy that introns play a crucial role in regulating gene expression (Ma et al., 2016). Nine genes, including *atpF*, *ndhA*, *ndhB*, *tRNA-CGA*, *tRNA-CGU*, *tRNA-UAA*, *tRNA-UAC*, *tRNA-UGC*, and *tRNA-UUU*, are shared by the three wild perennial *Hordeum* species and contain only one intron, while one gene, *ycf3*, contains two introns, which is unique to *H. brevisubulatum* and *H. violaceum* (**Supplementary Table 3**). In addition, the *ycf3* gene in the cultivated *Hordeum* species contains two introns (Middleton et al., 2013). Therefore, we contemplated that the absence of *ycf3* gene introns in *H. bogdanii* is unusual. Previous research has suggested that a species that a lack of gene introns in a species may indicate that it has taken on additional functions in diverse areas such as protease, RNA polymerase, and ribosomal pathways (Hakobyan et al., 2021).

### 4.2 Repeat sequences, RSCU, and PR2-plot analysis

Cp SSR in population genetics is considered a valuable molecular marker owing to its traits of matrilineal inheritance and low recombination frequency; gene insertion or deletion is also frequent in Cp SSRs (Xiao et al., 2019; Zong et al., 2019). Scattered repetitive sequences (SRS) and SSR of three wild perennial *Hordeum* species were analyzed and compared in the present study. The total number of SRS and cpSSRs in *H. bogdanii*, *H. brevisubulatum*, and *H. violaceum* were 231, 220, 218 and 182, 176, 176, respectively. *H. bogdanii* showed significantly different results from other two species, possibly due to their relatively close phylogenetic relatedness. In addition, the results of the study of *Secale sylvestre* (Skuza et al., 2022) and *Spartina maritima* (Rousseau-Gueutin et al., 2015) suggested that related species usually have similar SSR loci. Remarkably, most of the SSRs of the three *Hordeum* species are mononucleotides repeats dominated by poly-A or poly-T. This SSR phenomenon has not only been reported in the cp genomes of the Poaceae family (*Phalaris arundinacea* and *P. aquatica*) (Xiong et al., 2020) but also in other angiosperm families, such as *Hibiscus rosa-sinensis* (Abdullah et al., 2020), *Firmiana* (Abdullah et al., 2019), and *Taenia* (Yang et al., 2014).

During the translation of mRNA into proteins, there is an uneven frequency of synonymous codon usage called RSCU (Tyagi et al., 2020). In our study, 90.62% of codons with RSCU > 1 preferentially select A/U as the third nucleotide site, which is much higher than those ending with G/C, with similar results in many angiosperms such as *Nicotiana otophora* (Asaf et al., 2016), *Oryza minuta* (Sajjad et al., 2017), and *Medicago sativa* (Tao et al., 2016). The preference for A/U-ending codons is a common feature among most angiosperms and may be associated with certain evolutionary processes (Wang et al., 2023). PR2-plot analysis is essential for exploring codon bias. If the values of G_3_/(G_3_+C_3_) and A_3_/(A_3_+T_3_) are equal to 1, codon bias is completely influenced due to base mutation pressure; if both values are equal to 0, it is entirely because of natural selection (Wen et al., 2016). The majority of genes in our study had G_3_/(G_3_+C_3_) values greater than 0.5 and A_3_/(A_3_+T_3_) values lower than 0.5, indicating a bias towards G and T nucleotides in the third codon position, possibly due to a combination of natural selection and base mutations (Chen et al., 2021).

### 4.3 Sequence divergence

In the process of natural mutation, the probability of point mutation (SNP) is normally greater than that of frameshift (In/Del) (Raes and Van de Peer, 2005). As previously stated, the results of the cp genomes of the three *Hordeum* demonstrated that most mutations supported this conclusion. Interestingly, these mutation sites were concentrated in the intergenic or LSC region. The number of SNPs and In/Dels was significantly higher between *H. bogdanii* vs *H. brevisubulatum* and *H. bogdanii* vs *H. violaceum* compared with *H. brevisubulatum* vs *H. violaceum*. The reason may be that *H. bogdanii* was phylogenetically more distant from *H. brevisubulatum* and *H. violaceum*. Notably, no In/Dels were detected in the IR regions of *H. brevisubulatum* vs *H. violaceum*, suggesting that IR regions were the most conservative in the four-part structure (LSC, SSC, and IRa/IRb) of the cp genome, which warrants further exploration (Ravi et al., 2008). Pi, which is one of the standards that estimate the degree of nucleotide sequence variation and provide greater insight into the genetic variation to reflect complex changeable selection pressures in species and population levels (Namgung et al., 2021). Five genes with relatively high Pi values (Pi ≥ 0.1) were identified in the cp genomes, including *tRNA-UGC*, *tRNA-UAA*, *tRNA-UUU*, *tRNA-UAC*, and *ndhA*. These mutation hotspots can serve as a basis for further development of barcode molecular markers and phylogenetic analysis of the genus *Hordeum*.

The cp genomes of the 10 *Hordeum* species were analyzed for sequence variant and collinearity of using mVISTA and MAUVE procedures, respectively. The results indicated that the cultivated species, *H. vulgare*, were relatively conservative compared with the other wild related species. The wild plants undergo rapid molecular evolution due to which they form hotspot regions more frequently that are mainly located in the non-coding region of the LSC (Peng et al., 2021). Similar observations have been reported with *Morella rubra* (Liu et al., 2017) and three *Cardiocrinum* species (Lu et al., 2016). Notably, a series of hotspots regions were discovered, which mainly concentrated on *tRNA*-*GGU* ~ *tRNA*-*GCA*, *tRNA*-*UGU* ~ *ndhJ*, *psbE* ~ *rps18*, *ndhF* ~ *tRNA-UAG*, etc. Repeated conversions of genes between IRa and IRb regions may be a key factor responsible for generating these hotspots (Park et al., 2019). Collinearity analysis is generally a crucial strategy to determine the degree of cp genome variation (Liu et al., 2018). Collinearity analysis demonstrated that no rearrangement was detected in the cp genomes of the ten *Hordeum* species. However, there were significant differences were observed based on the cp genomes size, genotype, and expansion or contraction of IR boundaries.

As plants continue to evolve, the IR boundary can expand or contract due to the insertion or deletion of certain genes in the IR or SC region, which are the main factors contributing to cp genome size variation (Li et al., 2020). Here, the junction sites of the IR/SC region of the six cp genomes were analyzed using an online IRSCOPE software. In addition to the two annual *Hordeum* species (*H. vulgare* and *H. spontaneum*), no significant gene expansion, contraction, or loss was detected in the LSC/IRs/SSC boundary of the remaining four wild perennial *Hordeum* species (*H. bogdanii*, *H. brevisubulatum*, *H. violaceum*, and *H. jubatum*). This could be related to the fact that annual species have a more rapid evolutionary rate compared to perennial species (Duchene and Bromham, 2013). The length of the SSC region of *H. vulgare* was relatively smaller, mainly because the *ndhH* gene spanned the SSC/IRa region with 966 bp, which was the smallest compared with the other four wild perennial *Hordeum* species, located in the SSC region. Furthermore, the sites of genes *trnH* and *rps19* of *H. vulgare* changed significantly compared with those of the other *Hordeum* species. Besides, the *rpl22* gene only existed in the LSC region of *H. vulgare*, suggesting that it was replicated. This phenomenon may be attributed to the continuous domestication of the cultivated species, *H. vulgare*, leading to genetic changes through natural selection (Suoi et al., 2016). Therefore, the variation of the IR boundary and can be useful for phylogenetic studies of *Hordeum* species.

### 4.4 Phylogenetic relationships

The cp genome is quite conservative in sequence and structure, and the homology of molecular characters is easier to determine, thus it is a useful tool for constructing plant phylogeny (Yang et al., 2022). We conducted a phylogenetic analysis based on 31 Poaceae species (28 have been published and cp genomes of 3 *Hordeum* species were sequenced in the current study), with *Saccharum spontaneum* and *Sorghum bicolor* as the outgroups. The result showed that *H. bogdanii* has a further distance from *H. brevisubulatum* and *H. violaceum*. However, Jonathan et al. (Jonathan and Blattner, 2015) established a phylogenetic tree of these three *Hordeum* species based on the nuclear single-copy genome sequence analysis and demonstrated that they are clustered into a group. There may be two possible reasons for this difference. The first that the maternal ancestor of *H. bogdanii* is quite different from that of *H. brevisubulatum* and *H. violaceum*, and therefore it is hard to determine owing to relatively few reports on their matrilineal inheritance information. Another reason is the difference between the selected outgroups. In addition, although *H. brevisubulatum* (MT386010.1) has been published, the sequenced *H. brevisubulatum* in this study cannot be grouped into an identical subgroup. This may be because the former is a diploid or hexaploidy, while the latter is a tetraploid (Jakob and Blattner, 2006). Our findings provide valuable information for further investigation of the evolution trends of the cp genome in *Hordeum* species.

## 5 Conclusions

In summary, we sequenced and annotated the cp genomes of three *Hordeum* species (*H. bogdanii, H. brevisubulatum*, and *H. violaceum*) that exhibit a typical quadripartite structure. We then compared them to the cp genomes of two annual species, including one cultivated species (*H. vulgare*) and one wild species (*H. vulgare* subsp. *spontaneum*), as well as other five wild *Hordeum* species have been previously published. The results demonstrated that the cp genome of *H. vulgare* was more conserved although it contains a greater number of genes. Two mRNA genes, *ycf3* and *ycf4*, were not identified in *H. bogdanii*, of which *ycf3* contains two introns. Genes *trnG-UUC*, *trnI-GAU*, *rps12*, and *rps16* that are specific to only two annual *Hordeum* (*H. vulgare*, and *H. vulgare* subsp. *spontaneum*) and may be closely related to the regulation of *Hordeum* growth. Five highly polymorphic genes (*tRNA-UGC*, *tRNA-UAA*, *tRNA-UUU*, *tRNA-UAC*, and *ndhA*) and a series of hotspot regions, which mainly concentrated on *tRNA*-*GGU* ~ *tRNA*-*GCA*, *tRNA*-*UGU* ~ *ndhJ*, *psbE* ~ *rps18*, *ndhF* ~ *tRNA-UAG*, etc., were identified. These findings lay the foundation for further development of barcode molecular markers and phylogenetic analysis of *Hordeum* L. In addition, based on the phylogenetic tree analysis, *H. brevisubulatum* and *H. violaceum* were classified into the same group and were found to be relatively close phylogenetic relatives as compared with *H. bogdanii*. Finally, the present study highlights the degree of variation between wild and cultivated, as well as annual and perennial *Hordeum* species, providing insights into phylogenetic evolution and population genetics in the genus *Hordeum*.

## Data availability statement

The datasets presented in this study can be found in online repositories. The names of the repository/repositories and accession number(s) can be found below: https://db.cngb.org/, CNS0491101, https://db.cngb.org/, CNS0491102, https://db.cngb.org/, CNS0491103.

## Author contributions

SY and CN: Conceptualization, methodology, validation, formal analysis, investigation, data curation, writing – original draft, writing – review and editing, visualization. These authors contributed equally to this work and share the first authorship. YL and XM: Writing – review and editing, supervision, project administration, and funding acquisition. SJ, TL, JZ and JP: Investigation, resources, and writing – review and editing. WK and WL: Formal analysis, investigation, and data curation. YX and YLX: Methodology, software, validation, and formal analysis. XL and QY: Writing – review and editing. All authors contributed to the article and approved the submitted version.
