# Supplementary material for: Complete chloroplast genomes of three wild perennial Hordeum species from Central Asia: genome structure, mutation hotspot, phylogenetic relationships, and comparative analysis
Source: Front Plant Sci. 2023 Jul 24;14:1170004. doi: 10.3389/fpls.2023.1170004 (PMC10405828; doi:10.3389/fpls.2023.1170004)
Supplement: Supplementary file 1 [file DataSheet_1.pdf]

Supplementary Material

Complete Chloroplast Genomes of Three Wild Perennial Hordeum Species from Central Asia: Genome Structure, Mutation Hotspot, Phylogenetic Relationships, and Comparative Analysis

Shuai Yuan[1,2†], Cong Nie[1†], Shangang Jia[3], Tianqi Liu[1], Junming Zhao[1], Jinghan Peng[1], Weixia Kong[1], Wei Liu[1], Wenlong Gou[2], Xiong Lei[2], Yi Xiong[1], Yanli Xiong[1], Qingqing Yu[1], Yao Ling[1]*, and Xiao Ma[1]*

[1]College of Grassland Science and Technology, Sichuan Agricultural University, Chengdu, China，

[2]Sichuan Academy of Grassland Sciences, Chengdu 611743, China

[3]College of Grassland Science and Technology, China Agricultural University, Beijing, China

*** Correspondence:**
Xiao Ma
maroar@126.com

Yao Ling

ly9729752@163.com

[†] These authors have contributed equally to this work and share first authorship

1 Supplementary Data

The original contributions presented in the study are publicly available. The raw sequencing data were deposited into the China National GeneBank DataBase (CNGBdb) with the accession number CNS0491101, CNS0491102, and CNS0491103.

2 Supplementary Figures and Tables

2.1 Supplementary Figures

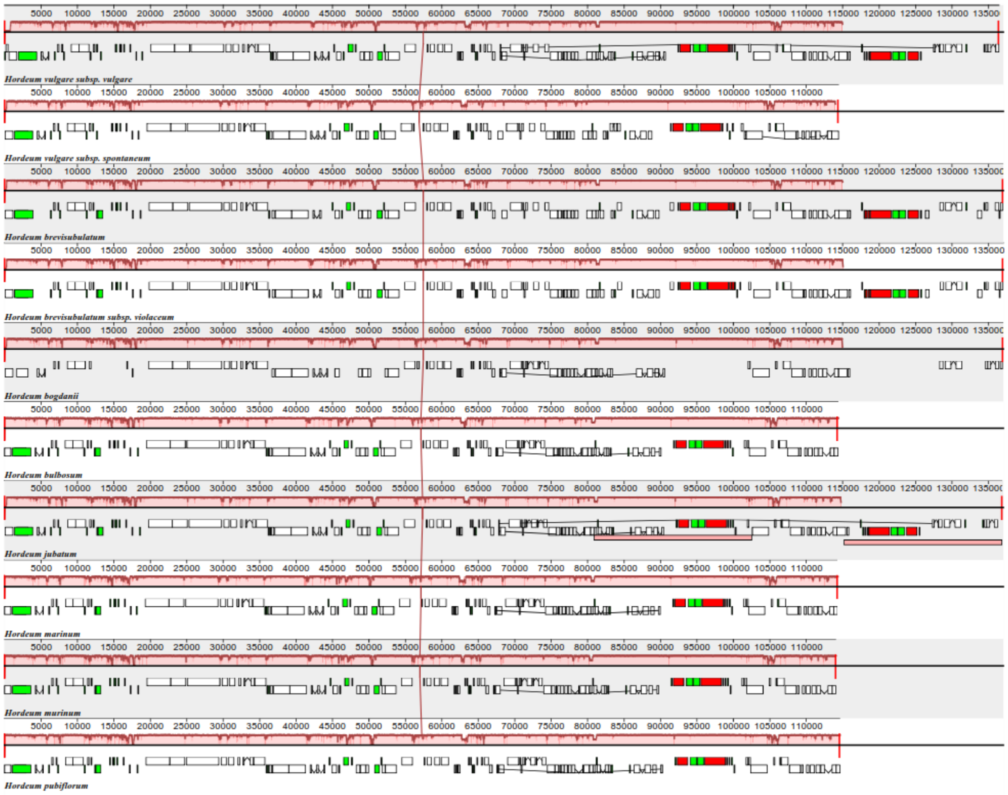

Supplementary Figure 1. Synteny comparison of ten *Hordeum* chloroplast genomes with the reference of *H. vulgare subsp. spontaneum* using Mauve.

Supplementary Table 1 Source information of three *Hordeum* species

Species	NPGS	GenBank Accession
H. bogdanii	PI 499498	CNS0491101
H. brevisubulatum	PI 531770	CNS0491102
H. violaceum	PI 531775	CNS0491103

Supplementary Table 2 Sampled Gramineae species along with their Genebank accession codes of cp genomes in this study.

Species	GenBank accession code
Hordeum vulgare	KT962228.1
Hordeum vulgare	MN171391.1
Hordeum vulgare	NC008590.1
Hordeum vulgare subsp. *spontaneum*	NC042692.1
Hordeum vulgare subsp. *spontaneum*	MH795067.1

Hordeum vulgare subsp. *spontaneum*	MN171385.1
Hordeum vulgare subsp. *spontaneum*	KC912688.1
Hordeum vulgare subsp. *trifurcatum*	MW017635.1
Hordeum vulgare subsp. *distichon*	MW531731.1
Hordeum vulgare subsp. *trifurcatum*	MW017635.1
Hordeum brevisubulatum	MT386010.1
Hordeum bogdanii	MW628890.1
Hordeum bogdanii	NC043839.1
Hordeum bulbosum	KY636105.1
Hordeum jubarum	KM974741.1
Hordeum pubiflorum	KY636108.1
Hordeum murinum	KY636107.1
Hordeum marinum	KY636106.1
Elymus kamoii	MW043483.1
Elymus sibiricus	NC058919.1
Elymus nutans	MG673520.1
Elymus dahuricus	NC049159.1
Triticum aestivum	KC912694.1
Triticum urartu	NC021762.1
Triticum monococcum	NC021760.1
Aegilops tauschii	NC022133.1
Aegilops cylindrica	NC023096.1
Agropyron cristatum	MN703671.1
Agropyron mongolicum	MH285848.1
Sorghum bicolor	NC008602.1
Saccharum spontaneum	LN896360.1

Supplementary Table 3 Location and length of intron-containing genes in the three *Hordeum* chloroplast genome.

Gene	Location	*H. bogdanii*			*H. brevisubulatum*			*H. violaceum*		
		Exon I (bp)	Intron I (bp)	Exon II (bp)	Exon I (bp)	Intron I (bp)	Exon II (bp)	Exon I (bp)	Intron I (bp)	Exon II (bp)
atpF	LSC	158	823	409	158	819	409	158	817	409
ndhA	SSC	550	1026	539	550	1037	539	550	1032	539
ndhB	IRA	777	712	756	777	712	756	777	712	756
ndhB	IRB	777	712	756	777	712	756	777	712	756
tRNA-CGA	LSC	32	660	63	32	659	63	32	659	63
tRNA-CGU	IRA	32	787	59	32	788	59	32	788	59
tRNA-CGU	IRB	33	785	60	33	786	60	33	786	60
tRNA-UAA	LSC	36	564	51	36	562	51	36	557	51
tRNA-UAC	LSC	39	579	54	39	579	54	39	579	54
tRNA-UGC	IRA	37	811	36	37	811	36	37	811	36
tRNA-UGC	IRB	38	809	37	38	809	37	38	809	37
tRNA-UUU	LSC	39	2478	37	39	2477	37	39	2477	37
ycf3	LSC				228	724	159	228	724	159

Supplementary Table 4 Summary of polymorphisms detected among three *Hordeum* chloroplast genomes

	H. bogdanii vs. *H. brevisubulatum*					*H. bogdanii vs. H. violaceum*					*H. brevisubulatum vs. H. violaceum*				
Gene	In/Del	Tn	Tv	Nonsyn	Total	In/Del	Tn	Tv	Nonsyn	Total	In/Del	Tn	Tv	Nonsyn	Total
atpA			3		3			5	2	5			2	2	2
atpB	1		2		3	1		2		3	-	-	-	-	-
atpF			2	2	2			2	2	2	-	-	-	-	-
atpI	-	-	-	-	-			1		1			1		1
ccsA	1		3		4	1		3		4	-	-	-	-	-
cemA		1	2	2	3			2	1	2		1		1	1
infA			2		2			1		1			1		1
matK			2	2	2			3	2	3			1		1
ndhA		1	3	1	4		1	3	1	4	-	-	-	-	-
ndhC			1		1			1		1	-	-	-	-	-
ndhD			2		2			2		2	-	-	-	-	-
ndhE			1		1			1		1	-	-	-	-	-
ndhF			6		6			8	2	8	1		2		3
ndhG			3		3			2		2			1		1
ndhH		1	3	1	4		1	3	1	4			2		2
ndhJ			1		1			1		1	-	-	-	-	-
ndhK		2	2	1	4		2	2	1	4	-	-	-	-	-
petA			4	2	4			3	1	3			1	1	1
petB			4		4		1	4	1	5			1	1	1
psaA		1	5	1	6		2	4	1	6		1	1		2
psaB			2		2			2		2			1		1
psaJ	1		1		2	1		1		2	-	-	-	-	-
psbA			2		2			2		2	-	-	-	-	-
psbB			2		2			1		1	-	-	-	-	-
psbC		1	3	2	4		1	3	2	4	-	-	-	-	-
psbK			1	1	1			1	1	1	-	-	-	-	-
rbcL		1	11		12		1	10		11	1		3		4
rpl16			1		1			1		1	-	-	-	-	-
rpl22	-	-	-	-	-			1		1			1		1
rpl23			2		2			2		2	-	-	-	-	-
rpoA			2	1	2			2	1	2	-	-	-	-	-
rpoB			4		4			4		4	-	-	-	-	-
rpoC1			7	1	7			7	1	7	1				1
rpoC2		1	11	5	12		1	12	5	13			3	2	3
rps18			1		1			1		1	-	-	-	-	-
rps19			2	2	2	-	-	-	-	-			2	2	2
rps32	1				1	1				1	-	-	-	-	-
rps2	-	-	-	-	-	-	-	-	-	-	1				1
rps3		1	1	1	2		1	1	1	2	-	-	-	-	-
rps8			2	1	2			2	1	2	-	-	-	-	-
ycf1			1		1			1		1	-	-	-	-	-
Subtotal coding	4	10	107	26	121	4	11	107	27	122	4	2	23	9	29
Subtotal noncoding	105	51	197	-	353	108	55	191	-	354	29	17	37	-	83
Total	109	61	304	26	474	112	66	298	27	476	33	19	60	9	112

Note: Tn, Transition; Tv, Transversion; In/Del, insertion or deletion
